# Characterization of Microbes Involved in Anaerobic Fermentation of Various Jiaosu

**DOI:** 10.1155/ijfo/7073400

**Published:** 2025-09-09

**Authors:** Ying Wang, Shuo Feng, Shi-min Liu, Zhi-sheng Zheng, Ling-xiao Wang, Xiu-fang Hu

**Affiliations:** ^1^ Zhejiang Province Key Laboratory of Plant Secondary Metabolism and Regulation, College of Life Sciences and Medicine, Zhejiang Sci-Tech University, Hangzhou, China, zstu.edu.cn

**Keywords:** *Acetobacter*, activities, components, enzyme, Jiaosu, *Lactobacillus*, yeast

## Abstract

Jiaosu is a fermentation product using microorganisms to transform organic materials into bioactive ingredients, which plays an important role in food industry, healthcare, and environmental protection. In this study, 37 microbial strains, including 22 lactic acid bacteria (LABs) and six yeast, were isolated from six different Jiaosu, with three genera (*Lactobacillus*, yeast, and *Bacillus*) shared by the five Jiaosu. The strains had the highest frequency of catalase (59.5%), followed by protease (56.8%), *β*‐glucosidase (54.1%), and lipase (40.5%), with lower frequency for cellulase (32.4%), pectinase (24.3%), and amylase (18.9%). Strains MZ3 and YT2 significantly inhibited pathogens *Escherichia coli* and *Staphylococcus aureus*, and strains MS2 and MZ3 inhibited *Ralstonia solanacearum*. DPPH free radical clearance was present in six strains including MZ1, MS4, and GJ2, while 25 strains showed over 60% clearance rate of ABTS, with the highest rate (96.2%) for strains GZ2, GS2, and MZ2. Nine strains were determined to have activities of SOD, POD, and CAT. All 37 strains had the ability to produce IAA, with higher IAA (25–135 *μ*g/mL) for seven strains. Strains MS2, MS4, MJ6, and MJ4 were found to produce siderophores, and strains YH4, JS7, YJ2, and GH1 produced GABA. Fourteen LAB strains had acidic culture solution (pH < 4), and four of which were confirmed to produce organic acids, GABA, and IAA based on LC‐MS. Therefore, functional strains were obtained from special Jiaosu, that is, organic acid–producing antagonist *Weissella confusa* MZ3, GABA, IAA‐producing antagonist *Pichia kudriavzevii* YJ2 and rich hydrolase–synthesis *Bacillus amyloliquefaciens* GC1 and *Lactobacillus plantarum* YS2, which might be potential microbial agents to improve the fermentation of Jiaosu.

## 1. Introduction

Fermentation is one of the oldest and most commonly used methods for making certain foods and food additives [1]. Jiaosu is a fermentation process that uses microorganisms to transform raw materials such as animals, plants, or fungi into products containing rich biological active ingredients [2]. As a multifunctional biocatalyst, Jiaosu plays an important role in the food industry, medical care, environmental protection, feed industry, and many other fields.

There are various types of Jiaosu depending on sources and functions. Classified by application field [3], the various types include edible Jiaosu, environmental Jiaosu, daily chemical Jiaosu, feed Jiaosu, and agricultural Jiaosu. According to the component raw materials, there are plant Jiaosu, fungal Jiaosu, animal Jiaosu, and mixed Jiaosu. Edible Jiaosu is usually used to prevent obesity‐related diseases; meet demand for nutrition, beauty, and health [4]; and regulate the microbial communities in the intestine [5]. Feed Jiaosu improves feed conversion and immune levels of poultry and significantly improves intestinal microflora [6, 7]. Environmental Jiaosu, made with fruit and vegetable wastes, is usually used in agriculture to improve the microbial diversity of rhizospheres, clean wastewater, and purify the air [8, 9]. Adding 10% environmental Jiaosu effectively reduces TSS, BOD, COD, SCOD, TVS, and other pollutants in dairy processing wastewater [10]. Agricultural Jiaosu has proved to be effective in improving soil quality, safely increasing plant disease and pest resistances, and promoting plant growth [11]. Therefore, improving the quality of Jiaosu might promote its utilization in many fields.

Microbes play great roles in the quality of Jiaosu. In the early fermentation stage, heterolactic bacteria are usually the dominant microbes, which help to start fermentation [12]. The dominant microbiota successively changed from poor acid resistance to *Lactobacillus* with strong acid resistance. Liang et al. [13] found that *Lactobacillus* grew rapidly at the initial stage of fermentation and then reached a stable fermentation level, while the number of *Vibrio*, *Halomonas*, *Leuconostoc*, and *Weissella* decreased. After 60 days of fermentation of alfalfa silage [14], the relative abundance of *Lactobacillus* continued to increase, while *Bacillus* continued to decrease. In apple Jiaosu [15], *Lactobacillus* and *Gluconobacter* were the dominant bacteria, and conjugative yeast and *Hansonomyces* were the dominant fungi. These results indicated that *Lactobacillus* is the dominant bacteria during the fermentation of Jiaosu.

The dominant microbes synthesize various enzymes for metabolism. Many enzymes, including amylase, protease, lipase, and cellulase, are found during the fermentation of Jiaosu. Some microorganisms degrade cellulose through cellulase to produce sugars, which are further fermented to improve raw material utilization [16, 17]. Rahman et al. [18] found that the highest amount of *α*‐amylase activity was found to be 1.782 units/mg, followed by lipase (0.355 units/mg) and protease (0.050 units/mg) in the Jiaosu produced by fruit and vegetable peel fermentation. In the saccharification stage of orange bagasse mixture fermentation [19], pectinase breaks down pectin, releasing sugars that are easily fermented by microbial fermentation and enhancing the release of flavor substances. Doan et al. [20] found that *Bacillus amyloliquefaciens* TKU050 was fermented on wheat bran, banana peel powder, orange peel powder, and other raw materials for 4 days, and the pectinase yield was the highest at 37°C (0.76 U/mL). In the process of sugarcane trash fermentation [21], *β*‐glucosidase activity increased at first and then decreased, and its activity was up to 59.3 U/g. These enzymes combinedly degrade and transform the raw materials into active components [22–26], such as organic acids, amino acids, vitamins, mineral elements, and active enzymes that variously exist in Jiaosu, which enhance its antioxidant capacity by reducing the pH of the system, clearing free radicals, and protecting cells from free radical damage.

Microbial agents are used in the fermentation of Jiaosu. Microbes are widely used as agents in fermentation, such as yeast in bread and beer manufacture [27], and *Lactobacillus* in yoghurt [28]. Yang et al. [29] found that *Lactobacillus acidophilus*, *Lacticaseibacillus casei*, and *Lactobacillus plantarum* promoted fermentation and improved the antioxidant and antibacterial ability of apple juice. The addition of lactic acid bacteria (LABs) effectively improves the fermentation quality [30]; *L. plantarum* inoculants were more effective in accumulating lactic acid and achieving good fermentation of sweet sorghum bagasse silage [31]. *Bifidobacterium*, *Saccharomyces cerevisiae*, and *Streptococcus thermophilus* are used in the fermentation of soft jujube kiwifruit Jiaosu [32], and three yeasts were used in banana fermentation [33]. However, multifunctional microbial agents are required to improve the fermentation of various Jiaosu, especially the strains with rich enzymes and active components.

In this study, microorganisms were isolated from various fermented Jiaosu including traditional Chinese dietary materials (ginger, garlic, and prickly ash), and their enzymes, antibacterial ability, and antioxidant activity were investigated. The purpose is to select special microbes that are rich in enzymes, active components, and functions and have high adaptability to acidic and anaerobic conditions, thereby enabling their use as microbial agents to promote the fermentation process and improve the quality of Jiaosu.

## 2. Methods and Materials

### 2.1. Preparation of Jiaosu

The various raw materials (ginger, garlic, prickly ash, peach, and kitchen garbage) were washed, air‐dried, and cut into pieces of 2–3 cm. The pretreated raw material was put into a plastic container with a sealed lid in a ratio of 1:3:10 with brown sugar, raw materials, and water. The mixture materials include ginger, garlic, prickly ash, and peach with the same ratio. The kitchen garbage constitutes equivalent fruit peels (banana, apple, and watermelon) and vegetable garbage (cabbage, lettuce, and cucumber leaves or peels). The containers were kept for fermentation in place with good air circulation at room temperature (20°C–35°C). Three replicates were set up for all six Jiaosu. After 30 days, the fermentation solution was sampled for microbial isolation for each Jiaosu.

### 2.2. Screening and Identification of Strains

Take 1.0 mL of each Jiaosu into a triangular flask containing 9.0 mL normal saline and shaken for 10 min, the solution was diluted to 10^−2^, 10^−3^, 10^−4^, 10^−5^, and 10^−6^. The dilutions of 10^−4^, 10^−5^, and 10^−6^ were selected, and 100 *μ*L of which was plated on the medium. The plates were cultured at 37°C for 48 h. Single colonies with different morphology of yeast, acetic acid bacteria, and LABs with large calcium‐soluble circles were selected and inoculated on YPD, MRS, and GYC media, respectively. After culturing at 37°C for 48 h, the purified strains were stored in −80°C.

The strains were molecularly identified. Targeting 16S rDNA (bacteria) or ITS (fungus) sequence, primers of 1492R/27F and ITS1/ITS4 were used to amplify the sequences, respectively. The amplified products were sequenced in Shenggong Bioengineering (Shanghai). The resulting sequences were compared using NCBI’s Nucleotide BLAST for identification. MEGA 7 software was used to generate a maximum likelihood phylogenetic tree using 1000 bootstraps.

### 2.3. Activities of the Strains

#### 2.3.1. Enzymatic Activity

Hydrolase: Hydrolase was qualitatively tested for all the strains and quantitatively assayed for the representative strains of the dominant genera.

Qualitatively test: Extracellular enzymes of the strains were determined by the diffusion method on plate culturing at 37°C for 2–3 days. Cellulase, pectinase, amylase, lipase, protease, catalase (CAT), and *β*‐glucosidase were detected according to the methods of Islam [15], Daskaya‐Dikmen et al. [34], Gilbert et al. [35], Vijayaraghavan and Vincent [36], Iwase et al. [37], and Srivastava et al. [38], respectively, with some slight modifications. The color and size of the transparent circles were observed to determine the activities of enzymes.

Quantitative assay: The strains were inoculated into the culture medium and incubated at 37°C for 48 h. The supernatant was obtained by centrifugation as the crude enzyme solution of the strain. The activities of amylase, lipase, and cellulase were determined by the method of Inan Bektas et al. [39], pectinase and CAT by the method of Hassan et al. [40], and *β*‐glucosidase activity by the method of González‐Hernández et al. [41].

Antioxidant enzymes: Take 3.0 mL of fresh bacterial liquid was firstly centrifuged at 8000 × g for 10 min, the supernatant was discarded, then the same volume of PBS was added, and the suspension was resuspended at 60% magnification. The solution was then ultrasonicated for 3 s at intervals of 3 s, and then ultrasonicated in an ice bath for 20 min until the bacteria were completely broken down. Finally, the bacterial solution was centrifuged at 8000 × g for 10 min at 4°C, and the supernatant was used for subsequent detection.

Superoxide dismutase (SOD) was determined by the nitroblue tetrazole (NBT) method [42] with absorbance measured at 560 nm. Peroxidase (POD) was determined according to the method of Ashraf et al. [43] with a few modifications. The CAT was determined by ultraviolet spectrophotometric method [44], and the absorbance was read at 240 nm and again at an interval of 180 s. Three replicates were set up for each test.

#### 2.3.2. Antagonistic Activity

The filter paper inhibition ring method [45] was used for antibacterial activity determination. The strains were inoculated into the medium and cultured at 37°C and 220 r/min for 24 h. The bacterial solution was adjusted to OD_600nm_ = 1.0. A small amount of 100 *μ*L of activated pathogen was evenly spread on the Petri dish. Three pieces of sterilized filter paper were placed at appropriate positions in three directions equal distance from the center of the plate, and 10 *μ*L of the tested bacteria droplets were aspirated on the filter paper. Three groups of replicates were carried out, and the strains were cultured in 37°C incubator for 48 h to observe the growth and inhibition zone of strains.

#### 2.3.3. Antioxidant Activities

##### 2.3.3.1. Scavenging of 2,2‐Diphenyl‐1‐Picrylhydrazyl (DPPH)

The DPPH scavenging capacity of the bacterial solution was determined by the method of Brand‐Williams et al.’s[46] method, with minor modifications. One milliliter of the probiotic suspensions to be tested was added into the reaction system, followed by 1 mL 0.1 mmol/L DPPH of anhydrous ethanol solution, which was thoroughly mixed and oscillated for 30 min at room temperature without light and then centrifuged at 6000 r/min for 10 min. The supernatant was then taken, and its absorbance was measured at 517 nm. Vitamin C was used as a positive control. The DPPH free radical clearance rate is calculated, as shown in the following formula [47]:

DPPH free radical clearance rate/%=Control A−sample AControl A ×100.

##### 2.3.3.2. Scavenging of 2,2 ^′^‐Azino‐Bis (3‐Ethylbenzothiazoline‐6‐Sulfonic Acid) (ABTS)

The samples for ABTS radical scavenging assay were carried out according to the method of Urbonavičienė et al. [48], and the absorbance was measured at 734 nm. The positive control was Trolox (6‐hydroxyl‐2,5,7,8‐tetramethylcyclohexane‐2‐carboxylic acid). The free radical clearance rate of the sample was calculated through the following formula: ABTS^+^ scavenging effect (*%*) = 1 − (*A*_1_ − *A*_2_)/*A*_0_, where *A*_0_ is the absorbance of the control group and *A*_1_ is the absorbance of the sample.

### 2.4. Active Components

#### 2.4.1. Indole Acetic Acid (IAA)

The Salkowski method [49] was employed for determining the production of IAA. The strains were cultured in liquid medium at 37°C with 220 r/min for 24 h and centrifuged to obtain the supernatant for measurement of absorbance. The production of IAA was calculated based on the standard curve.

#### 2.4.2. *γ*‐Aminobutyric Acid (GABA)

GABA is a nonprotein amino acid widely distributed in plants, animals, and microorganisms [50]. Using the disk diffusion method, a sterilized circular filter paper was placed on a plate, and 7 *μ*L of the tested bacteria was dropped onto the filter paper and incubated for 48 h at 37°C. The strains that produce GABA appeared green.

#### 2.4.3. Siderophores

Siderophores are secondary metabolites with a high affinity for Fe^3+^ and are usually secreted by microorganisms to chelate iron in the environment [51]. To determine siderophores, 7 *μ*L of bacterial suspension was added to chrome azurol S (CAS) medium [52] and cultured at 30°C for 5 days. A yellow halo indicated the production of siderophores, and the diameter of the transparent circle was measured.

### 2.5. Detection of Metabolic Substances

To determine the proper point for detection of metabolic substances, the pH value was investigated during culturing of the strains. The strains were inoculated in 50 mL liquid medium and cultured with 220 rpm at 37°C for 48 h, and pH was measured every 6 h. The pH value was determined by a pH meter (PB‐10 pH, Sartorius, Beijing, China).

At the earliest stable pH value, the culture solution of strains was sampled for detection of metabolic substances using LC‐MS. The culture solution was centrifuged at room temperature at 12,000 rpm for 10 min, and the supernatant was filtered using 0.22 *μ*m filter membrane The filtered solution was transferred into a clean chromatographic bottle for measurement. Chromatographic column: Waters ACQUITY UPLC HSS T3 C18 1.8 *μ*m, 2.1 mm∗100 mm; Mobile phase A was acetonitrile, while Mobile Phase B was 0.2% phosphate buffer [53]. For gradient elution, the elution ratio was acetonitrile: water = 3.5*%* : 96.5*%*, flow rate was 0.8 mL/min, and sample size was 20 *μ*L. The specific parameters of the mobile phase are shown in Table 1, and the mass spectrum conditions are shown in Table 2.

**Table 1:** HPLC mobile phase gradient conditions.

**Time (min)**	**Velocity of flow (mL/min)**	**A (%)**	**B (%)**
0.0	0.8	95	5
11.0	0.8	10	90
12.0	0.8	10	90
12.1	0.8	95	5
14.0	0.8	95	5

**Table 2:** Mass spectrometry conditions.

**Argument**	**ESI+**	**ESI−**
Ion source voltage (V)	250	1500
Auxiliary gas flow rate (L/min)	8	8
Fragmentation voltage (V)	135	135
Ion source temperature (°C)	325	325
Sheath temperature (°C)	325	325
Sheath gas velocity (L/min)	11	11
Atomizing gas voltage (V)	40	40

### 2.6. Statistical Analysis

Each experiment was tested in triplicate. Data are expressed as the mean ± standard deviation (SD), with statistical significance set at *p* < 0.05. Statistical analysis was performed using SPSS Statistics 26 (IBM Corp., Armonk, NY, United States).

## 3. Results

### 3.1. Screening and Identification of Fermentation Strains

Microbes were isolated from six fermented Jiaosu that were based on different raw materials. In total, 37 strains were collected on three specific media (YPD, MRS, and GYC), including 14 strains from MRS medium, 12 strains from YPD, and 11 strains from GYC (Figure 1a,b). For the different types of Jiaosu, four strains were obtained from peaches Jiaosu, nine strains from ginger Jiaosu, seven strains from garlic Jiaosu, five strains from kitchen garbage Jiaosu, and six strains from mixture Jiaosu and prickly ash Jiaosu, respectively. For the microbial genera, six strains belonged to yeast, that is, three *Pichia* and three *Saccharomyces*, while 31 strains belonged to bacteria, including 22 LABs (i.e., 19 *Lactobacillus* and three *Weissella*), one *Acetobacter*, three *Bacillus*, and five other genera. On the species level, LABs play a leading role in the fermentation process, mainly manifesting as *L. plantarum*, *Lactobacillus casei* , and *Weissella confusa*.

Figure 1Phylogenetic trees based on sequences of (a) ITS and (b) 16S rDNA and Venn diagrams on levels of (c) genus and (d) species between different Jiaosu.

**Figure:**
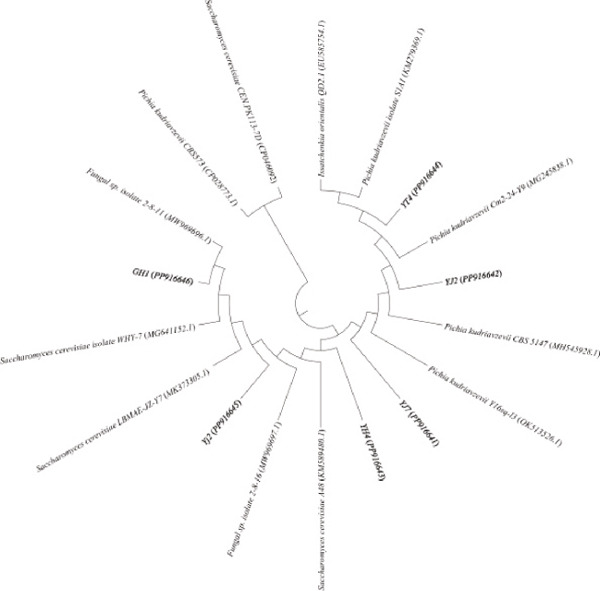
(a)

**Figure:**
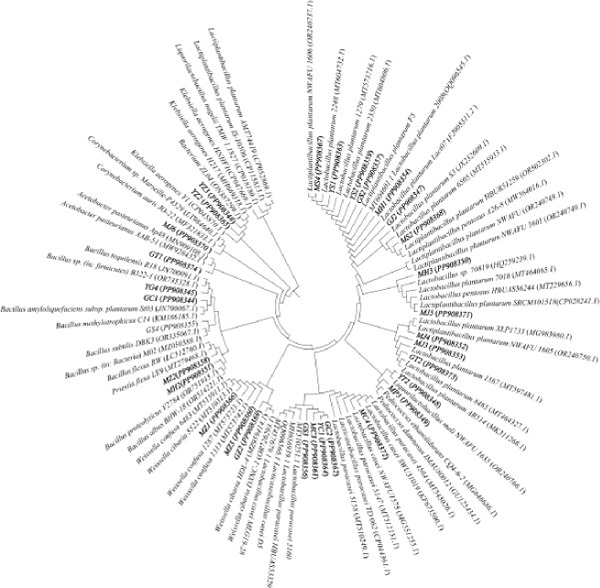
(b)

**Figure:**
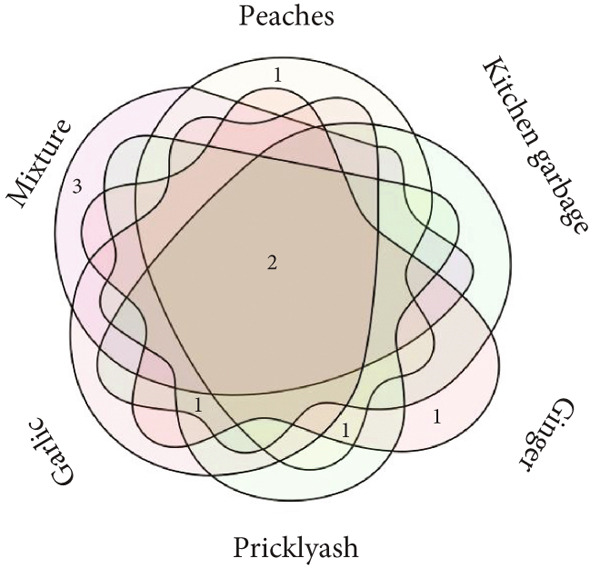
(c)

**Figure:**
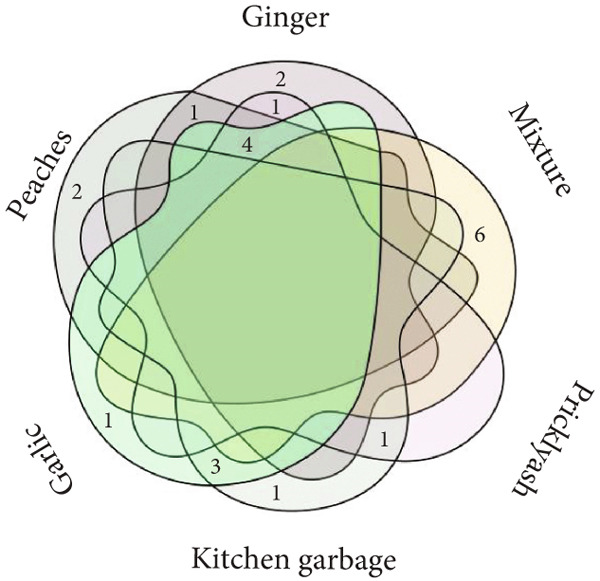
(d)

Based on the Venn diagram (Figure 1c,d), three genera (*Lactobacillus*, yeast, and *Bacillus*) were shared by the five Jiaosu except for prickly ash or mixture, respectively; one genus (*Bacillus* sp.) existed in the four Jiaosu except for peaches and mixture; and one genus existed in the three Jiaosu, including peaches, ginger, and mixture. In addition, there were two (*Klebsiella aerogenes* and *Priestia flexa*), one (*Corynebacterium auris*), and one (*Acetobacter*) genus in the single Jiaosu of mixture, ginger, and peaches, respectively. As for the species, all five Jiaosu had special species of bacteria except for prickly ash Jiaosu, while there was one species in the two Jiaosu, such as peaches/ginger, ginger/prickly ash, and garlic/kitchen garbage. Only three genera, *Lactobacillus*, yeast, and *Bacillus*, were shared by the four Jiaosu except for mixture and kitchen garbage Jiaosu.

### 3.2. Activities of Strains

#### 3.2.1. Enzyme Activities

Seven enzymes were tested to investigate the biodegradabilities of the strains from the six Jiaosu. To investigate the enzyme profile in each Jiaosu, the positive enzymes were calculated as the enzyme frequency of the strains from each Jiaosu. As shown in Figure 2a and Table 3, the strains from garlic Jiaosu had the highest frequency of hydrolase (53.06%), followed by prickly ash (47.62%), mixture (45.24%), and kitchen garbage (42.86%), with the lowest frequency (26.98%) for ginger Jiaosu. As for the types of hydrolases, there were seven enzymes from the strains of prickly ash, garlic, and kitchen garbage Jiaosu; seven enzymes except for pectinase or amylase from the strains of ginger and mixture Jiaosu; and five enzymes except for cellulase and pectinase from peaches. Amylase, *β*‐glucosidase, protease, lipase, and CAT were present in all six Jiaosu, indicating their roles in the major metabolism of 30 days Jiaosu. Among them, the frequency of CAT occurrence was between 50% and 75%, and the frequency of lipase was between 33.3% and 60%. However, the frequencies of protease and *β*‐glucosidase varied between the different Jiaosu, with the highest frequencies (85.71% and 42.85%) in garlic Jiaosu and with the lowest frequencies in peaches (50% and 50%) and ginger (22.22% and 55.56%) Jiaosu, respectively. Cellulase and pectinase mainly existed in the strains from garlic (57.14% and 42.86%), mixture (50% and 50%), and prickly ash (33.33% and 33.33%) Jiaosu.

Figure 2(a) Enzyme frequency of the strains in each Jiaosu and (b) genus frequency of the strains for each enzyme.

**Figure:**
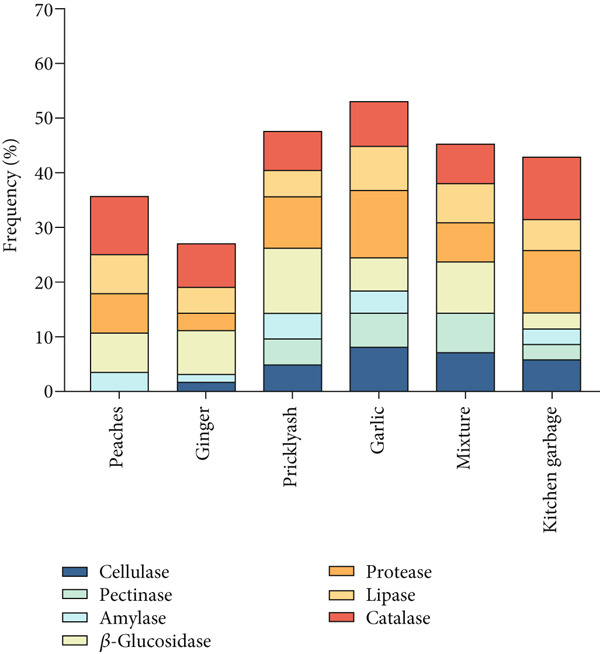
(a)

**Figure:**
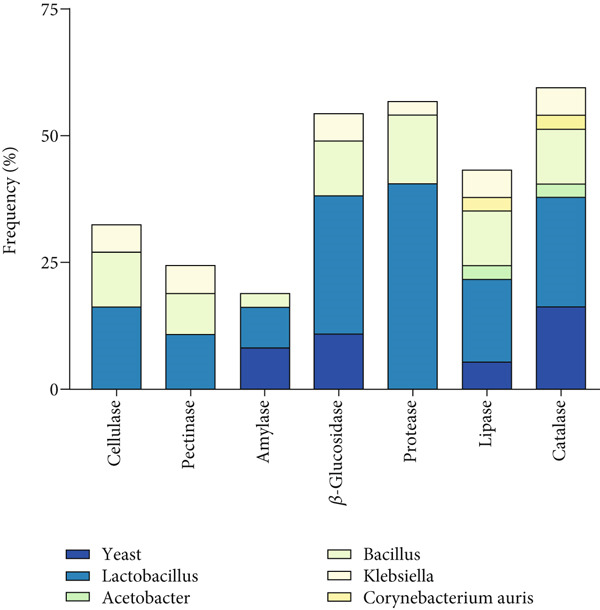
(b)

**Table 3:** Bacteria screened from different raw materials and their hydrolase activities.

**Source**	**Strain**	**Cellulase**	**Pectinase**	**Amylase**	***β*-Glucosidase**	**Protease**	**Lipase**	**Catalase**
Peaches	*Pichia kudriavzevii* YT4	−	−	+++	++	−	−	+++
	*Acetobacter pasteurianus* GT1	−	−	−	−	−	++	+++
	*Lactobacillus plantarum* GT2	−	−	−	−	+	−	−
	*Liquorilactobacillus nagelii* YT2	−	−	−	++++	++++	+++	++

Ginger	*Pichia kudriavzevii* YJ7	−	−	−	++	−	−	+++
	*Pichia kudriavzevii* YJ2	−	−	+	+++	−	−	++
	*Saccharomyces cerevisiae* Yj2	−	−	−	−	−	++	++
	*Lactobacillus plantarum* MJ3	−	−	−	+++	−	−	−
	*Lactobacillus plantarum* MJ4	−	−	−	+++	−	−	−
	*Lactobacillus plantarum* MJ5	−	−	−	+++	−	−	−
	*Lactobacillus plantarum* GJ2	−	−	−	−	+++	−	++
	*Corynebacterium auris* MJ6	−	−	−	−	−	++	+++
	*Bacillus tequilensis* GJ4	++	−	−	−	++++	+++	−

Prickly ash	*Saccharomyces cerevisiae* YH4	−	−	++	−	−	+	++++
	*Saccharomyces cerevisiae* GH1	−	−	−	+++++	−	−	++++
	*Lactobacillus plantarum* MH1	−	−	−	+++	++	−	−
	*Lactobacillus plantarum* GH2	++	+	−	+	+	−	−
	*Lactobacillus plantarum* MH3	−	−	+++	++	+++	−	−
	*Bacillus amyloliquefaciens* MH2	+	+++	−	+++++	++	++	+

Garlic	*Lactobacillus plantarum* MS2	++	+	−	−	+++	−	+
	*Lactobacillus plantarum* MS4	++	−	−	−	+++++	−	−
	*Lactobacillus plantarum* GS2	−	−	−	−	+++	−	+
	*Lactobacillus paracasei* GS3	−	−	−	++++	−	+++	−
	*Lactobacillus plantarum* YS1	−	−	−	+++	+++	+++	+
	*Lactobacillus plantarum* YS2	+	++	+++	−	+++	+	+
	*Bacillus subtilis* GS4	++	+++	++	+	++	+++	++

Kitchen garbage	*Lactobacillus paracasei* MC3	−	−	−	−	+++	−	++
	*Lactobacillus paracasei* GC2	+	−	−	−	−	−	+
	*Lactobacillus paracasei* MC4	−	−	−	−	++	−	−
	*Lactobacillus casei* YC1	−	−	++	−	+++	++	+++
	*Bacillus amyloliquefaciens* GC1	++	+++	−	++++	+++	+++	+

Mixture	*Klebsiella aerogenes* YZ2	++	++++	−	++++	−	+++	++
	*Klebsiella aerogenes* YZ3	++	++	−	+	+++	+++	++
	*Weissella confusa* MZ1	+	−	−	−	++	−	−
	*Weissella confusa* MZ3	−	+++	−	−	−	−	−
	*Weissella cibaria* GZ2	−	−	−	++++	−	++	−
	*Priestia flexa* MZ2	−	−	−	+++	+++	−	++

*Note:* Activity intensity by “−” to “**+++++**” level, where a “−” means no activity and “**+++++**” means strong activity.

To investigate the genus profile in the 37 strains, the genus frequency was calculated for each enzyme. As shown in Figure 2b, the strains had the highest frequency of CAT (59.5%), followed by protease (56.8%), *β*‐glucosidase (54.1%), and lipase (43.2%), with the lowest frequencies for amylase (18.9%), pectinase (24.3%), and cellulase (32.4%). Concerning the types of microbes, all six types of microbes had lipase and CAT, four types of microbes (*yeast*, *Lactobacillus*, *Bacillus*, and *Klebsiella*) had *β*‐glucosidase, while three types of microbes had cellulase, pectinase, amylase, and protease. *Lactobacillus* and *Bacillus* had all seven of the enzymes, and *Klebsiella* had six enzymes, except for amylase. Yeast had four enzymes, such as amylase, *β*‐glucosidase, lipase, and CAT, while *Acetobacter* and *C. auris* only showed lipase and CAT with moderate activity. For the special strains (Table 4), *β*‐glucosidase production was higher in five strains, of which MH2 and GH1 had the most significant production. For protease activity, *Lactobacillus* accounted for 85%, represented by strains YT2, GJ2, and MS4. Strain YT4 had the strongest activity of amylase. The reactions of YH4 and GH1 for CAT were the fastest and strongest. Strains GT2, GS4, YZ2, and YZ3 had strong lipase capacity.

**Table 4:** The metabolites identified through LC‐MS.

**Strain**	**Retention time (min)**	**Molecular formula**	**Measured value of excimer peak (m/z)**	**Excimer peak theoretical value (m/z)**	**Compound**
GT1	0.796	C_4_H_9_NO_2_	104.14	103.14	Gamma‐aminobutyric acid
	1.354	C_2_H_4_O_2_	61.05	60.0521	Acetic acid
	0.817	C_10_H_9_NO_2_	176.1919	175.19	IAA

GZ2	0.786	C_4_H_9_NO_2_	104.14	103.14	Gamma‐aminobutyric acid
	0.793	C_4_H_6_O_5_	135.0291	134.09	Malic acid
	1.559	C_4_H_4_O_4_	117.08	116.08	Fumaric acid
	14.77	C_3_H_6_O_3_	91.08	90.08	Lactic acid
	1.281	C_10_H_9_NO_2_	176.1919	175.19	IAA

MZ3	9.460	C_4_H_9_NO_2_	104.14	103.14	Gamma‐aminobutyric acid
	3.089	C_3_H_4_O_3_	89.06	88.062	Pyruvic acid
	14.77	C_3_H_6_O_3_	91.08	90.08	Lactic acid
	0.786	C_6_H_8_O_6_	177.1321	176.12	Ascorbic acid
	0.867	C_7_H_6_O_3_	139.1287	138.12	Salicylic acid
	0.817	C_10_H_9_NO_2_	176.1919	175.19	IAA

GS3	0.765	C_4_H_9_NO_2_	104.14	103.14	Gamma‐aminobutyric acid
	1.229	C_7_H_6_O_2_	123.1293	122.14	Benzoic acid
	1.292	C_4_H_6_O_5_	135.0291	134.09	Malic acid
	14.77	C_3_H_6_O_3_	91.08	90.08	Lactic acid
	1.250	C_10_H_9_NO_2_	176.1919	175.19	IAA

MS4	0.765	C_4_H_9_NO_2_	104.14	103.14	Gamma‐aminobutyric acid
	1.292	C_4_H_6_O_5_	135.0291	134.09	Malic acid
	2.490	C_6_H_12_O_7_	197.12	196.1553	Gluconic acid
	3.057	C_3_H_4_O_3_	89.06	88.062	Pyruvic acid
	14.77	C_3_H_6_O_3_	91.08	90.08	Lactic acid

Four strains of different genera, *Bacillus amyloliquefaciens* GC1, *L. plantarum* YS2, *K. aerogenes* YZ2, and *Pichia kudriavzevii* YJ2, were selected as representatives to quantify the activities of different enzymes. As shown in Table 5, the strain GC1 showed the most enzymes except for amylase with higher activities; the strain YS2 possessed high amylase; moderate cellulase, pectinase, and protease; low CAT; and no *β*‐glucosidase; the strain YZ2 had five enzymes except for amylase and protease, while strain YJ2 had only amylase, *β*‐glucosidase, and CAT. The quantitative results were similar to those qualitative activities for the four strains.

**Table 5:** Quantitative activities of enzymes for four representative strains (units per milliliter).

**Strain**	**Cellulase**	**Pectinase**	**Amylase**	***β*-Glucosidase**	**Protease**	**Lipase**	**Catalase (U/gprot)**
GC1	125.44 ± 5.98	131.3 ± 3.15	—	156.98 ± 11.54	102.54 ± 6.88	102.9 ± 6.98	388.92 ± 9.67
YS2	68.98 ± 4.55	106.86 ± 9.68	116.14 ± 5.78	—	98.47 ± 5.23	51.45 ± 5.52	140.95 ± 15.25
YZ2	113.54 ± 0.89	163.64 ± 4.55	—	143.25 ± 13.36	—	95.1 ± 2.26	320.46 ± 3.69
YJ2	—	—	59.53 ± 1.78	126.84 ± 5.68	—	—	333.33 ± 7.73

#### 3.2.2. Antibacterial Activity

The antibacterial potential of the 37 strains against three pathogenic bacteria (*Escherichia coli*, *Staphylococcus aureus*, and *Ralstonia solanacearum*) was measured. As shown in Figure 3, strains MS2 and MZ3 had significant inhibitory effects on *R. solanacearum* (Figure 3a), while five strains, including MZ3, YJ2, YJ7, YT2, and YJ1, had inhibitory effects on *E. coli* (Figure 3b). For *S. aureus* (Figure 3c), five strains, including MZ3, MS4, YT2, MJ6, and YJ2, showed inhibitory effects. *W. confusa* MZ3 showed inhibitory effects on the three pathogens, with the largest inhibition zone against *E. coli*. Compared with the average size of the inhibition zones, it was obvious that the antagonistic activities of the strains against *R. solanacearum* and *E. coli* were much higher than those against *S. aureus*.

Figure 3Antipathogen activities of the strains (a) *Ralstonia solanacearum*, (b) *Escherichia coli*, and (c) *Staphylococcus aureus.*

**Figure:**
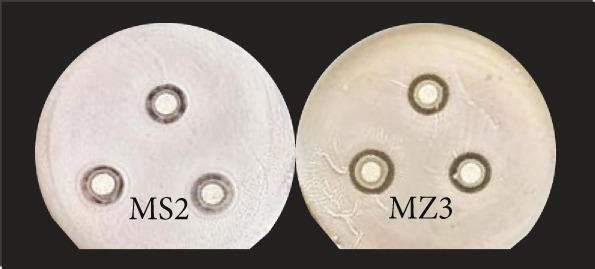
(a)

**Figure:**
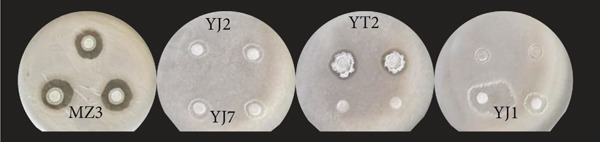
(b)

**Figure:**
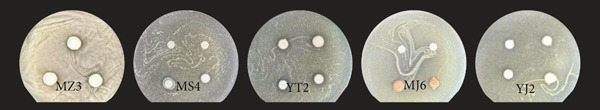
(c)

#### 3.2.3. Antioxidant Activity

Antioxidant activities of DPPH and ABTS were determined in the tested strains. As shown in Figure 4a,b, the higher DPPH free radical clearance rate was present in strains *L. plantarum* MZ1, MS4, and GJ2, followed by strains YJ7, MH3, and GC2, while strains GT1, YZ2, and YS1 had the lowest free radical scavenging rate. For ABTS, 25 strains showed over 60% clearance rate, with the highest rate (96.2%) for strains GZ2, GS2, and MZ2, while strains YJ7, YJ2, and MS4 had the lowest clearance rates (20.3%).

Figure 4Antioxidant activities of the strains (compared with MS2,  ^∗^ *p* < 0.05,  ^∗∗^ *p* < 0.01, and  ^∗∗∗^ *p* < 0.001). (a) DPPH, (b) ABTS, and (c) antioxidant enzymes.

**Figure:**
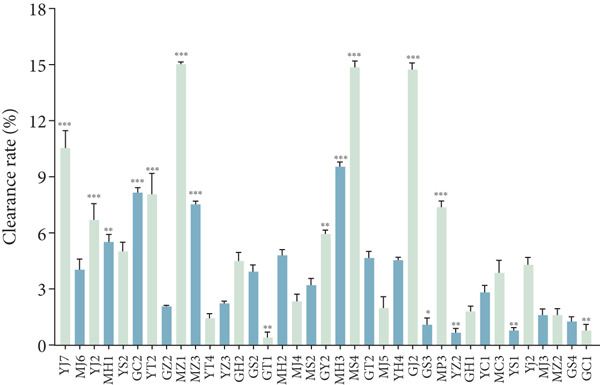
(a)

**Figure:**
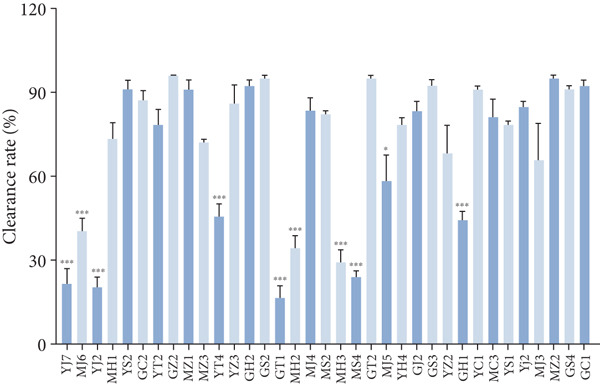
(b)

**Figure:**
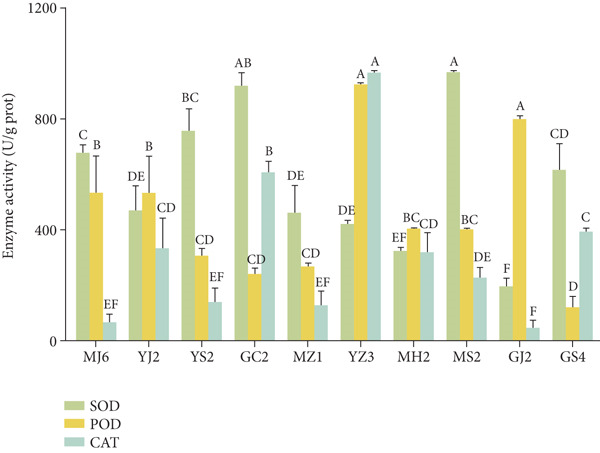
(c)

Based on enzyme activities, nine strains were selected to test the activity of antioxidant enzymes. As shown in Figure 4c, all nine strains had activities of SOD, POD, and CAT, with higher activity for SOD, followed by CAT and POD. The value of SOD activity ranged from 195.98 to 968.71 U/gprot, with the highest for MS2 (968.71 U/gprot), YS2 (847.97 U/gprot), and GC2 (942.16 U/gprot). The highest POD activity was present in strain YZ3 (925.00 U/gprot), followed by MJ6 (533.33 U/gprot) and YJ2 (533.33 U/gprot), while the highest CAT activity was present in strain YZ3 (966.67 U/gprot), followed by GC2 (606.67 U/gprot) and GS4 (393.33 U/gprot). The higher activities of the three antioxidant enzymes were found for strain GC2, while the lowest activities were found for strain GJ2. The other strains had higher activity of single SOD, POD, or CAT.

### 3.3. Active Components

#### 3.3.1. IAA, GABA, and Siderophores

The strains were tested for the production of IAA. As shown in Figure 5a, all the strains had the ability to produce IAA, among which, seven strains produced higher IAA (25–135 *μ*g/mL), with the highest production for strains YZ2 (137.85 *μ*g/mL), YJ2 (121.21 *μ*g/mL), and YZ3 (97.58 *μ*g/mL), while five strains, such as GZ2, MJ4, MJ5, GH1, and YJ2 produced the lowest IAA (less than 1 *μ*g/mL). The other strains produced medium IAA levels, with the range of 2–40 *μ*g/mL.

Figure 5Active components produced by the strains. (a) Production of IAA (compared with MS2,  ^∗^ *p* < 0.05,  ^∗∗^ *p* < 0.01, and  ^∗∗∗^ *p* < 0.001). (b) Ability to produce GABA. (c) Synthesis of siderophores. (d) Ratio of transparent circles for siderophores (*D*/*d*).

**Figure:**
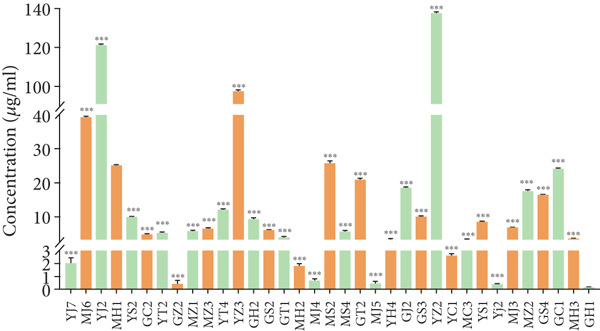
(a)

**Figure:**
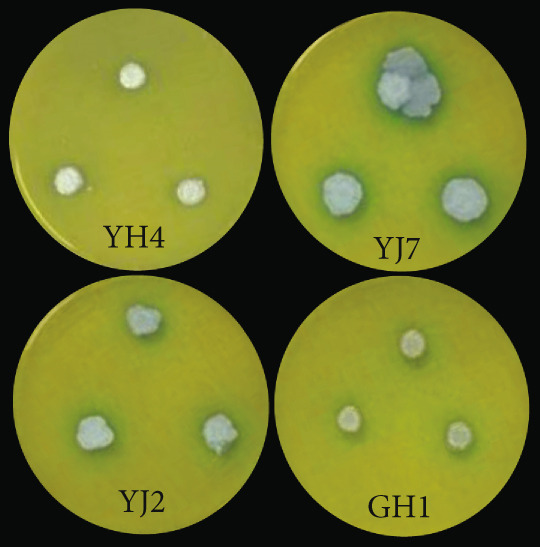
(b)

**Figure:**
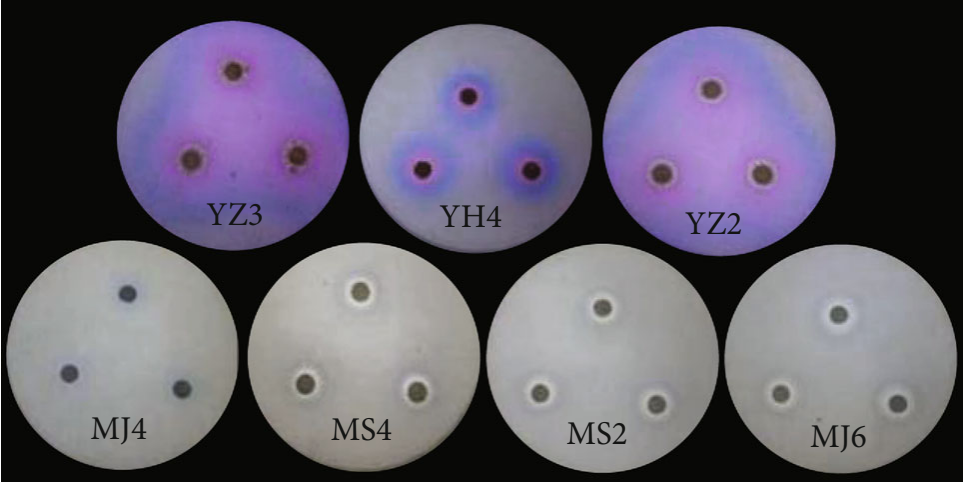
(c)

**Figure:**
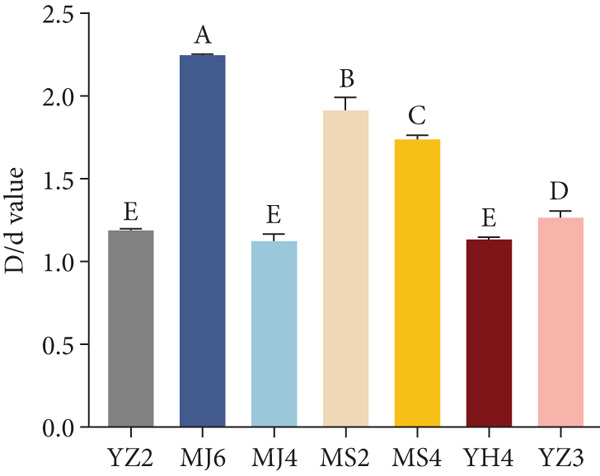
(d)

The production of GABA was also investigated for all the strains. As can be seen from Figure 5b, the four strains YH4, JS7, YJ2, and GH1 produced a blue–green change, indicating the production of GABA, with the highest production for strain YJ7, followed by strains YJ2, GH1, and YH4.

For siderophores, it was observed that some microbes produced siderophores. As shown Figure 5c, seven strains were found to form hydrolytic transparent rings. Strains MS2, MS4, MJ6, and MJ4 formed obvious pale yellow lysospheres on CAS medium, while strains YH4, YZ2, and YZ3 showed purple transparent circles. These observations indicated that two different types of iron carriers might be produced, which require further evidence to substantiate. Strains MJ6 and MS2 had higher *D*/*d* values (Figure 3d), indicating higher production of siderophores.

#### 3.3.2. Organic Acid Production

The pH of the fermentation solutions was determined. As shown in Figure 6a, the pH values of 11 typical species of microbes were measured during culturing, and six strains (MZ3, GT1, GZ2, GH1, MS4, and MH2) showed a continuous decrease of pH to about 4, while the other strains showed a complex trend and maintained higher pH levels (about 6.5). All the strains had relatively stable pH at 72 h post culture. Therefore, the pH of all the strains was tested at 72 h postculture. For the results (Figure 6b), 17 strains such as yeast and *Bacillus* showed higher pH values (6 or higher), while 14 strains had lower pH values (about 4) including LABs and *Acetobacter*. The other six strains had pH 4–6. The strains with lower pH values might produce organic acids. Therefore, the strains with lower pH in their fermentation culture were selected to detect the metabolites using LC‐MS.

Figure 6Changes of pH in the liquid culture of the strains (compared with MS2,  ^∗^ *p* < 0.05,  ^∗∗^ *p* < 0.01, and  ^∗∗∗^ *p* < 0.001). (a) Growth curves of some representative strains. (b) pH values of the strains.

**Figure:**
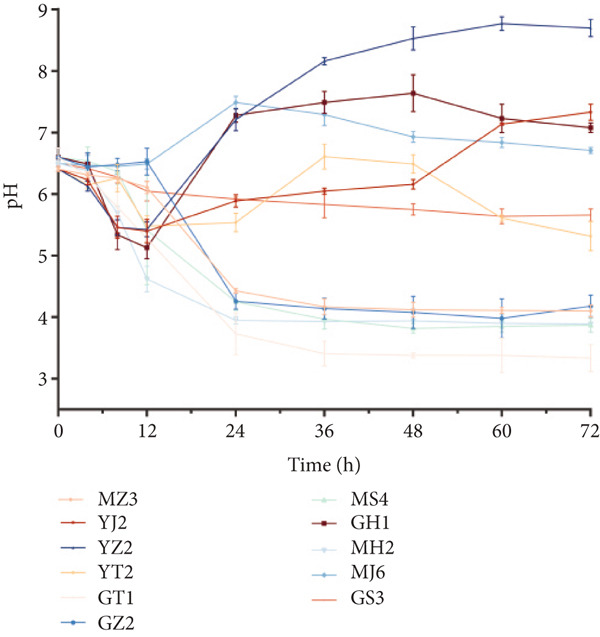
(a)

**Figure:**
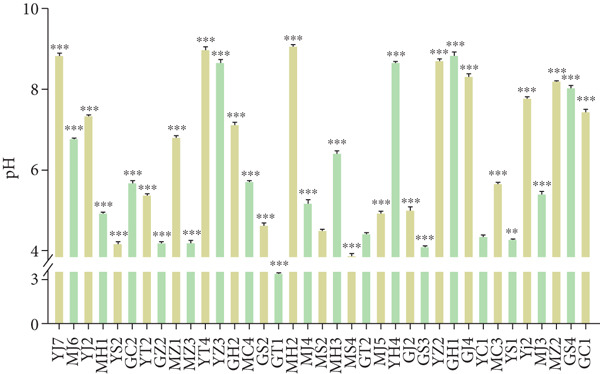
(b)

For the production of organic acids (Figures 7a, 7b, 7c, 7d, and 7e), based on the LC‐MS results, a variety of metabolites were detected and identified, such as GABA, IAA, and riboflavin. Strain MS4 produced the most organic acids (lactic acid, malic acid, gluconic acid, and pyruvate), but no GABA was detected. Strains MZ3 and MS4 produced pyruvate, and strains GZ2, GS3, and NS4 produced malic acid. Strain MZ3 produced ascorbic acid and salicylic acid. All five strains produced IAA.

Figure 7Positive ion peak mass chromatograms of the strains. (a) Strain GT1, (b) strain GZ2, (c) strain MZ3, (d) strain GS3, and (e) strain MS4.

**Figure:**
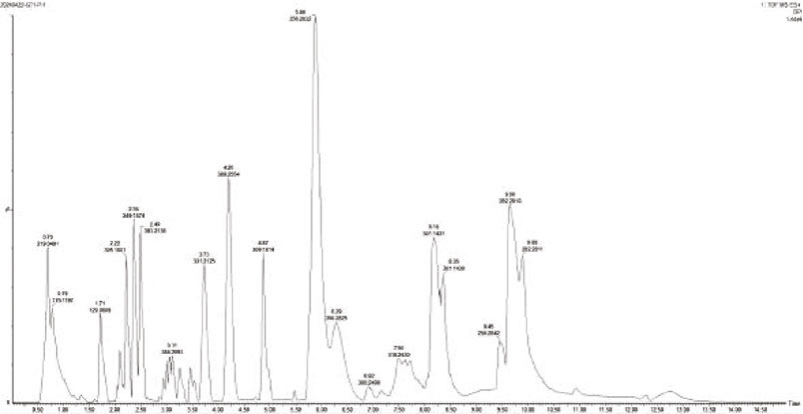
(a)

**Figure:**
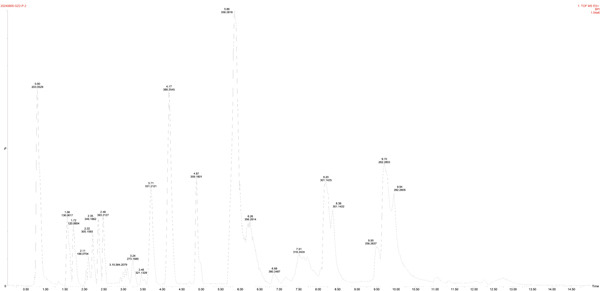
(b)

**Figure:**
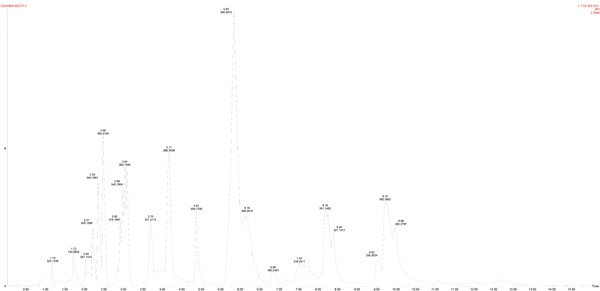
(c)

**Figure:**
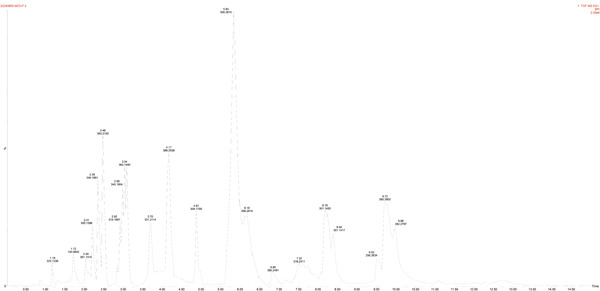
(d)

**Figure:**
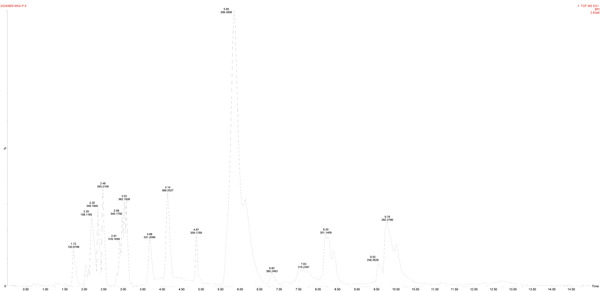
(e)

## 4. Discussion

Fermentation is widely used in the transformation of food, herbal, and organic garbage. During fermentation, rich microbes reproduce, and various enzymes are involved in converting nutrients into valuable metabolites; therefore, microbes and enzymes play vital roles in fermentation [54]. In this study, microbes were isolated from six types of Jiaosu that were fermented from different raw materials, and the microbes synthesized various components, including enzymes, organic acids, and hormones, which endowed the strains with potential functions.

The microbes involved in the fermentation of Jiaosu shifted with the nature of the raw materials and the stage of fermentation. The different raw materials contained various original components and microbes, which accordingly induced different microbes to reproduce during special stages. Therefore, the diversity and abundance of microbes involved in the fermentation fluctuated with the raw materials and process. During the early stage of fermentation, the microbial communities are richer and varied with the raw materials. Padrós et al. [55] detected more than 17 kinds of bacteria, mainly *Aerococcus viridans*, *Enterococcus casseliflavus*, *Lactobacillus parafarraginis*, *Marinilactibacillus* sp., *Vibrio furnissii*, and *Weissella paramesenteroides* in the fermentation of Spanish‐style green olives. During the fermentation of wolfberries [56], the 10‐day samples were dominated by 57.92% *Lactobacillus*, 14.23% *Lactococcus*, 12.08% *Weissella*, 9.45% *Enterococcus*, and 4.41% *Pediococcus*, while the 20‐day samples were mainly dominated by 77.28% *Lactobacillus* and 19.39% *Enterococcus*. Firmicutes (*Lactobacillus*, *Weissella*, *Acetobacter*, and *Enterococcus*) and Proteobacteria (*Enterobacterium*, *Pseudomonas*, and *Relella*) were the main dominant bacterial phyla in the early stages of Jiaosu, while the relative abundance of *Lactobacillus* increased significantly as the fermentation continued [57]. As the fermentation progressed, the pH values began to drop to 5.0 or lower, with acid‐resistant microorganisms, such as *Acetobacter* and *Lactobacillus*, gradually becoming the dominant microorganisms [58, 59]. In all fermented foods and beverages, LABs are the dominant microbiota and are considered to be the most critical microorganisms in promoting the beneficial effects of fermented food products [60]. In this study, *Lactobacillus* (22 strains) are the major microbes identified from the 30‐day fermented Jiaosu with different materials, followed by *Saccharomyces* (six strains). LAB play a leading role in the fermentation process, mainly manifest as *L. plantarum*, *L. casei*, and *W. confusa*. However, the genera *Lactobacillus*, yeast, and *Bacillus* were common among the five Jiaosu except for prickly ash or mixture, respectively. There were unique genera in some Jiaosu, such as *K. aerogenes* and *P. flexa* in mixture, *C. auris* in ginger, and *Acetobacter* in peaches. Therefore, various types of Jiaosu shared similar dominant genera of *Lactobacillus* in 30‐day fermentation, with unique species in Jiaosu with special materials of ginger, peaches, or the mixture. In light of the distinctive properties of the special materials, such as the hot (ginger essential oil and gingerol) [61, 62] of ginger and the astringency (phenolic compounds) [63] of peaches, the unique bacteria might adapt to the special components, which can be a particular resource of microbes for the fermentation of special materials.

Microbial synthesis varied the enzymes present in the different Jiaosu. During the fermentation of Jiaosu, different microorganisms reproduce and produce various enzymes to break down organic matter; therefore, the types and activities of enzymes accordingly change with the raw materials and the fermentation process [64]. During the fermentation of tomato Jiaosu, the activities of amylase and cellulase increased gradually to the maximum of 79.289 and 38.180 U/g, respectively; the activity of protease fluctuated in the early stage of fermentation and increased significantly, while pectinase and lipase increased first and then decreased, with their maximum activities at 20 days (3.733 U/g) and 60 days (0.856 U/g), respectively [65]. After 3 months of fermentation, the amylase activity of tomato and mango waste solution was 0.7 and 0.3 U/mL, respectively, while the protease activity of pineapple and tomato wastes was 0.75 and 0.1 U/mL, respectively [66]. During the fermentation of pineapple and citrus wastes (6:4) [67], the maximum activities of amylase, lipase, and protease were 57.289, 43.987, and 76.024 U/mL, respectively. Among the 137 strains isolated from dairy products, 61.3% strains had proteolytic activity, while 50.3% strains showed lipolytic activity [68]. *Lactobacillus* had significantly higher *β*‐glucosidase activity, with the highest activity for strain ACCC11095 (400.70 *μ*U/mL) [69]. In this study, most of the strains from the 30‐day Jiaosu had strong proteinase, *β*‐glucosidase, and lipase activities, while fewer strains had cellulase, pectinase, and amylase. In addition, the strains isolated from garlic showed the most enzymes, followed by the strains from prickly ash and the mixture. These results indicated that more enzymes were present at the early fermentation stages, while fewer enzymes, especially *β*‐glucosidase and protease, mainly existed at the late stages.

Rich metabolites were produced by microbes in the Jiaosu. In this study, organic acids, hormones, and vitamins were produced. IAA and SA were determined from the products of the strains, with the highest yields for strains YZ2, YZ3, and YJ2. IAA has been identified in numerous plant‐associated bacteria and some fungi [70–72]. *Lactobacillus* has seldom been reported to synthesize IAA, while it was found to be related to the increase of IAA in the gut and thus activate aryl hydrocarbon receptor to enhance the gut barrier function [73]. Petkova et al. [74] found that the IAA synthesis levels of yeasts were *Zygosaccharomyces bailii* YE1 (0.463 mg/L), *S. cerevisiae* YD5 (0.512 mg/L), and *Saccharomyces kudriavzevii* YSW1 (0.625 mg/L). In this study, most *Lactobacillus* and yeasts produced IAA (*Pichia pastoris*, *S. cerevisiae*, *L. plantarum*, and *L. casei*), and the highest production was by *P. kudriavzevii* YJ2, with the exceptions of strains *L. plantarum* MJ4, MJ5, *S. cerevisiae* GH1, and *Weissella cibaria* GZ2. In addition, yeasts including *S. cerevisiae* YH4, GH1 *P. kudriavzevii* YJ7, and YJ2 produced higher levels of GABA, while *W. cibaria* GZ2, MZ3, *L. paracasei* GS3, and *Acetobacter pasteurianus* GT1 produced lower levels of GABA. Some *Lactobacillus* and yeasts produce GABA based on high glutamic acid decarboxylase activity, usually with the yield in the ranges of 0.015–30 g/L by *Lactobacillus* and 0.015–10 g/L by yeasts [75].

Organic acids are the main metabolites of Jiaosu. In this study, some strains, including *Acetobacter*, some *Bacillus*, and all LAB, had pH values of 3–6 in the fermentation, indicating the production of organic acids. Based on LC‐MS, *A. pasteurianus* GT1 produced acetic acid; *L. plantarum* MS4, *L. paracasei* GS3, *W. confusa* MZ3, and *W. cibaria* GZ2 produced malic acid and fumaric acid; and strain MS4 produced more acids, such as succinic acid, butyric acid, and pyruvic acid. During the first 2–5 days of fermentation, the reducing sugars are rapidly converted to lactic acid, formic acid, acetic acid, propionic acid, and butyric acid [76]. Arun and Sivashanmugam [77] found that the concentrations of acetic acid, lactic acid, oxalic acid, malic acid, and citric acid were 11.12, 26.02, 44.81, 11.05, and 39.05 g/L in the waste Jiaosu on the 15th day of fermentation, respectively. After fermentation, pineapple, orange, and lemon waste Jiaosu all contained 80%–90% acetic acid, while banana waste Jiaosu contained 38% acetic acid and 42% butyric acid [78]. The pH value of Radix Astragalus, matrine, and Radix Bupleuri fermented for 90 days was 3.26, with acetic acid as the main organic acid (13.08 g/L), followed by propionic acid (0.96 g/L), butyric acid (0.63 g/L), and formic acid (0.02 g/L), respectively [11]. Three combinations of Jiaosu, namely, orange–papaya–watermelon (OPW), grapefruit–mango–pineapple (GMP), and durian–jackfruit–passion fruit (DJP) have the highest acetic acid (9.723 g/L) and lactic acid (9.958 g/L), with great increases of acetic acid in OPW (24.4 times), GMP (99.3 times), DJP (51.0 times), tartaric acid (15.1 times) in DJP, lactic acid in OPW (111 times), and DJP (109 times), as well as citric acid in GMP (22.3 times) and DJP (13.8 times) [79]. LAB metabolize sugars in the absence of oxygen to produce lactic acid, such as 6.08 g/L lactic acid by *Lactiplantibacillus plantarum* and 6.16 g/L by *L. casei* [80]. The acetic acid, lactic acid, and formate in LAB100 were dominant with 44.81, 17.11, and 7.90 g/L, respectively, while succinate in LAB690 was the highest with 20.83 g/L [51]. In the fermentation process of *S. cerevisiae*, the organic acid content showed a trend of increasing at first and then stabilizing, with the maximum value of 3.242 g/L [81]. Therefore, rich organic acids are formed in Jiaosu, which change by type and levels according to the particular raw materials, microbes, and fermentation process present.

Microbes produced antioxidant components. Significant scavenging of ABTS and DPPH was tested for the strains, reflecting their antioxidant activity. Park et al. [82] showed improved scavenging ability of DPPH and ABTS in Jiaosu with fruit as raw material. In the fermented guava juice, the incidence of DPPH increased from 64.1% to 83.1% [83]. Three types of Jiaosu from fruit and vegetable peel, citrus, noncitrus, and vegetable showed 64%, 53%, and 61% scavenging activity of DPPH, respectively [84]. Antioxidant enzymes are considered to be an important enzymatic antioxidant defense system in LAB [85]. *L. paracasei* NM‐12, *Enterococcus faecium* UM‐12 and NM‐11, and *Pichia fermentans* QY‐4 had effective antioxidant enzyme activities, with 4.846–9.105 U/mL SOD, 0.0140–0.1396 U/mL GPX, and 3.749–97.289 U/mL CAT [86]. Organic acids or extracellular polysaccharides secreted by *Lactobacillus* have metal ion chelating activity, which can reduce oxidative stress and enhance the antioxidant effect in synergy with antioxidant enzymes in the body [84, 85]. In addition, the enhancement of antioxidant function is partly attributed to the production of phenols and vitamins and other active compounds by microbes in Jiaosu [87]. In this study, antioxidant enzymes, organic acids, and ascorbic acids were tested from the strains, which might play great roles in their antioxidant activity. Therefore, enzymatic and nonenzymatic antioxidant activities endow the Jiaosu with great antioxidation potential.

Microbes produce antagonistic components. Strain MZ3 inhibited the growth of *E. coli*, *S. aureus*, and *Rella soliculosa*, while strains YT2 and YJ7 inhibited *E. coli* and *S. aureus*. Strains MS2, YJ1, YJ7, MS4, and MJ6 inhibited only one of the pathogens. LABs exert their antagonistic effects through a variety of mechanisms and components, including the production of bacteriocins such as lactic streptococci to inhibit the growth of pathogenic bacteria, the secretion of organic acids (such as lactic acid, acetic acid, and propionic acid) to reduce the environmental pH, and the production of metabolites (such as hydrogen peroxide and antimicrobial peptides) to inhibit the metabolic activities of pathogenic bacteria [88–91]. The good antibacterial activity of *Weissella* is mainly due to its production of antibacterial compounds, such as lactic acid and bacteriocins. Wang et al. [92] showed that 0.5% (*v*/*v*) lactic acid prevented the growth of pathogens, such as *Salmonella* sp., *E. coli* and *Listeria monocytogenes*. Tenea and Lara [93] proved that the bacteriocins produced by the *Weissella* Cys2‐2 inhibited the activity of *E. coli*, *Salmonella*, and *Shigella* strain MZ3 isolated in this study produces lactic acid, which can inhibit microorganisms by increasing the permeability of cell membrane, changing the intracellular osmotic pressure, and inhibiting the synthesis of intracellular macromolecules. In addition, LABs compete for nutrients and growth factors, interfere with pathogen virulence signaling systems, enhance intestinal epithelial barrier function, and produce exopolysaccharides to prevent pathogen adhesion and biofilm formation [94]. Siderophore can help microorganisms to obtain iron, while some antibacterial substances inhibit microbial growth by interfering with siderophore synthesis or competing with siderophore for iron [95] and enhance antibacterial effect. The antibacterial mechanism of strains MS2, MS4, and MJ6 may be related to lactic acid and siderophore, which need further evidence.

Obviously, common and special microbial strains with multiple functions were systematically screened from six Jiaosu, including strains producing rich hydrolase enzymes, active components, as well as antioxidation and antagonism (Table 6).

**Table 6:** Comparison between this study and previous studies.

**Item**	**Results in this study**	**Results from reference**
Microbial species	Common species: LABs, yeasts, and bacilli from six Jiaosu Special species: *Priestia flexa* and *Klebsiella aerogenes*, *Corynebacterium auris*, and *Acetobacter* from Jiaosu of mixture, ginger, and peaches, respectively	LABs, yeasts, and bacilli from fermentation of silage, fruit and vegetable, and bamboo shoot [13, 96]
Enzyme activity	Protease (56.8%) and *β*‐glucosidase (54.1%) dominate on 30‐day Jiaosu Higher enzymes in garlic Jiaosu	Early stage of fermentation: Amylase, cellulase, and protease Later stage of fermentation: Lipase, *β*‐glucosidase, and pectinase [33, 77, 97]
Active metabolites	Organic acids, GABA, ascorbic acid, salicylic acid, IAA, and siderophore	Organic acids: Lactic acid [80] and acetic acid [82]
Functions	Antioxidants: Scavenging ROS with SOD, CAT, and POD Antagonism: Inhibit pathogens	Single function: Bacteriostasis [98] or antioxidant [99]

## 5. Conclusion

In conclusion, genera of *Bacillus*, *Lactobacillus*, *Saccharomyces*, and *Pichia* were isolated from six Jiaosu, with higher activities of protease, CAT, and *β*‐glucosidase, as well as active products of organic acids, IAA, GABA, and siderophore. Four multifunctional strains were obtained, namely, organic acid producing antagonist *W. confusa* MZ3, GABA, IAA‐producing antagonist *P. kudriavzevii* YJ2, and rich hydrolase‐synthesis *Bacillus amyloliquefaciens* GC1 and *L. plantarum* YS2, which might be potential bacterial resources for application in fermentation of Jiaosu with various functions.

## Ethics Statement

Our study did not require ethical board approval because it did not contain human or animal trials. This research did not involve human participants and animals.

## Disclosure

We assure the integrity and quality of our research work. It is also stated that there is no plagiarism in this work, and all points taken from other authors are well cited in the text. This study is entirely independent and impartial.

## Conflicts of Interest

The authors declare no conflicts of interest.

## Funding

This study was funded by the Open Project of Zhejiang Provincial Key Laboratory of Agricultural Green Biomanufacturing Core Strain Improvement (2021DG700024‐KF202518) and the Scientific Research Fund of Zhejiang Sci‐Tech University (20042221‐Y).

## Data Availability

The original datasets generated from the study are available from the corresponding author upon reasonable request.
